# Ultrasonic radiomics-based nomogram for preoperative prediction of residual tumor in advanced epithelial ovarian cancer: a multicenter retrospective study

**DOI:** 10.3389/fonc.2025.1540734

**Published:** 2025-02-04

**Authors:** Shanshan Li, Qiuping Ding, Lijuan Li, Yuwei Liu, Hanyu Zou, Yushuang Wang, Xiangyu Wang, Bingqing Deng, Qingxiu Ai

**Affiliations:** ^1^ Department of Medical Ultrasound, The Central Hospital of Enshi Prefecture Tujia and Miao Autonomous Prefecture, Hubei Selenium and Human Health Institute, Enshi, Hubei, China; ^2^ Reproductive Medicine Center, The Central Hospital of Enshi Prefecture Tujia and Miao Autonomous Prefecture, Enshi, Hubei, China; ^3^ Department of Medical Ultrasound, The Ethnic Hospital of Enshi Tujia and Miao Autonomous Prefecture, Enshi, Hubei, China; ^4^ Department of Medical Ultrasound, The Maternal and Child Health and Family Planning Service Center of Enshi Tujia and Miao Autonomous Prefecture, En Shi, Hubei, China

**Keywords:** ultrasonic radiomics, ovarian cancer, predictive model, nomograms, residual tumor

## Abstract

**Objectives:**

To identify radiomic features extracted from ultrasound images and to develop and externally validate a comprehensive model that combines clinical data with ultrasound radiomics features to predict the residual tumor status in patients with advanced epithelial ovarian cancer (OC).

**Methods:**

The study included 112 patients with advanced epithelial OC who underwent preoperative transvaginal ultrasound. Of these, 78 patients were assigned to the development cohort and 34 to the external validation cohort. Tumor contours were manually delineated as regions of interest (ROI) on the ultrasound images, and radiomic features were extracted. The final set of variables was identified using LASSO (least absolute shrinkage and selection operator) regression. Clinical features were also collected and incorporated into the model. A combination model integrating ultrasound radiomics and clinical variables was developed and externally validated. The performance of the predictive models was assessed.

**Results:**

A total of 1,561 radiomic features and 18 clinical features were extracted. The final model included 10 significant ultrasound radiomic variables and 4 clinical features. The comprehensive model outperformed models based on either clinical or radiomic features alone, achieving an accuracy of 0.84, a sensitivity of 0.80, a specificity of 0.75, a precision of 0.88, a positive predictive value of 0.81, a negative predictive value of 0.86, an F1-score of 0.78, and an AUC of 0.82 in the external validation set.

**Conclusions:**

The comprehensive model, which integrated clinical and ultrasound radiomic features, exhibited strong performance and generalizability in preoperatively identifying patients likely to achieve complete resection of all visible disease.

## 1 Introduction

Ovarian cancer (OC) ranks among the most prevalent gynecological cancers, holding the position of the third most commonly diagnosed malignancy in the female reproductive system, surpassed only by cervical and endometrial cancers. Moreover, it exhibits the highest mortality rate within this category of cancers, posing a significant threat to women’s health (1). Because early symptoms are often nonspecific, the majority of patients are diagnosed at an advanced clinical stage, frequently presenting with localized or widespread pelvic and abdominal metastases. Despite initial treatment, recurrence rates and mortality remain high, with frequent development of drug resistance. As a result, the 5-year survival rate is below 40%, leading to a generally poor prognosis for these patients (2).

According to the International Federation of Obstetrics and Gynecology (FIGO), there are two main treatment strategies for advanced OC in stages IIIC-IV: (1) primary debulking surgery (PDS) followed by six cycles of postoperative platinum-based chemotherapy, and (2) for patients unlikely to achieve satisfactory tumor reduction, two to three cycles of neoadjuvant chemotherapy can be given before interval debulking surgery (IDS), followed by postoperative adjuvant chemotherapy, a strategy commonly referred to as “sandwich” therapy (3). The primary goal of both treatment approaches is to maximize tumor reduction, ideally leaving a residual tumor (RT) diameter of less than 1 cm, or achieving no visible residual tumor (R0). Maximal cytoreduction stands as a critical prognostic factor in the treatment of advanced OC, showing the most favorable outcomes following adjuvant chemotherapy.

Unfortunately, not all OC patients are suitable candidates for primary debulking surgery (PDS) aimed at achieving an R0 resection (4). For those with a low probability of attaining R0 resection, there is a consensus that surgical intervention should be avoided if incomplete resection (with residual tumor greater than 1 cm) is anticipated, as it has little benefit to patient survival and may lead to a high incidence of perioperative related diseases (3–5). Therefore, assessing the probability of a patient’s RT-resection during PDS prior to surgery is advantageous, as it supports the implementation of individualized treatment strategies.

In recent years, the field of imaging has made significant advancements, allowing for a more detailed depiction of tumor heterogeneity and providing valuable prognostic information (6). Various mathematical approaches have been applied to extract a vast array of radiomic features from medical images with high throughput, enabling clinicians to improve diagnostic accuracy and develop personalized, precision treatments (7, 8). Transvaginal ultrasound is a commonly utilized, cost-effective method for the clinical diagnosis of OC, and ultrasound radiomics has been increasingly employed in the study of various malignancies, including thyroid, cervical, liver, and OC (9–11). For example, Chiappa et al. utilized ultrasound radiomics to distinguish between malignant and benign ovarian tumors, highlighting its potential to enhance the preoperative evaluation of patients with ovarian masses and accurately identify those with OC (12). Thus, a comprehensive and unbiased assessment of ultrasound image features is essential (10).

This study seeks to assess the predictive significance of ultrasound radiomics and clinical factors in creating and validating a more reliable and generalizable preoperative model for forecasting RT status in patients with advanced epithelial OC. The goal is to standardize and simplify the process for gynecologists, enabling them to extract critical information from traditional diagnostic imaging more effectively and make informed decisions based on it.

## 2 Materials and methods

### 2.1 Study population

The study enrolled 112 patients with histologically confirmed FIGO stage III or IV OC diagnosed between January 2018 and June 2024. Of these, 78 patients from the Central Hospital of Enshi Tujia and Miao Autonomous Prefecture formed the development cohort, while 34 patients, recruited by collaborators at the Ethnic Hospital of Enshi Tujia and Miao Autonomous Prefecture, comprised the external validation cohort. The inclusion and exclusion criteria were consistent across both cohorts. The exclusion criteria included patients currently undergoing neoadjuvant chemotherapy, those lacking essential clinical or surgical data, individuals with poor image quality or significant image artifacts affecting visualization, and patients with a history of repeated biopsies. We established a standardized protocol to define dataset variables and outcomes, enabling the retrospective collection of data within the same time frame. Patients who met the inclusion criteria were divided into two groups: (1) the RT<1 group, comprising individuals with no visible gross residual tumor (RT) and a maximum tumor diameter of less than 1 cm; and (2) the RT≥1 group, which included patients with a maximum tumor diameter of 1 cm or greater (13). This retrospective study was approved by our institution’s ethics review board, with informed consent obtained from all participants.

### 2.2 Clinical information

Clinical data, including age, body mass index (BMI), parity, presence of hydrothorax, ascites, and ASA score, as well as the metastases in abdomen and pelvis (MAP) score, were collected. Laboratory findings such as perioperative platelet count, perioperative albumin levels, serum cancer antigen-125 (CA125), serum human epididymis protein 4 (HE-4) levels, and the neutrophil-to-lymphocyte ratio (NLR) were also obtained. Additionally, ultrasonic measurement characteristics such as maximum tumor diameter, arterial pulsatility index, resistance index, end diastolic flow rate, peak flow rate, and average flow rate were retrieved from the medical records.

The MAP score was assessed based on preoperative enhanced CT scans of the abdomen and pelvis, with two radiologists, blinded to intraoperative records, scoring and documenting the findings. The score was based on the Zhongshan Hospital rating scale for preoperative OC, which assessed lesions in various regions, including the diaphragmatic peritoneum, liver and kidney recesses, liver capsule, hepato-gastric space, spleen and stomach space, greater omentum (covering both the liver area and splenic curvature), mesentery, peritoneum, intestines, paracolic sulci, uterorectal space, uterine bladder space, and lymph nodes. Each identified lesion contributed 2 points, with the total score being the cumulative sum of all lesions. Any discrepancies in scoring were resolved through consensus.

### 2.3 Image segmentation

In accordance with the Institutional Review Board’s approved protocol, essential clinical data and ultrasound image locations were systematically documented in standardized electronic case report forms (CRFs) and collected within four weeks prior to the primary surgical intervention. The segmentation of images was conducted independently by two seasoned radiologists who were unaware of the patients’ tissue pathology. One of the radiologists, possessing around 12 years of experience, utilized the open-source ITK-SNAP software (version 3.8.0; www.itksnap.org) to manually delineate the regions of interest (ROIs) on the image slices. The Kappa consistency analysis was performed to evaluate discrepancies between two radiologists, and a Kappa value ≥ 0.85 was regarded as a good consistency.

### 2.4 Radiomics feature extraction

PyRadiomics (v.2.0.0; http://www.radiomics.io/pyradiomics.html) software was used to extract features from medical images (14). The process included importing manually delineated ROI images along with the original images into the PyRadiomics platform, where an internal feature analysis program was utilized to extract the relevant features. We adopted nonlinear intensity transformation on image voxels, Gaussian Laplace filter and Eight wavelet transform to obtain high-throughput features. Radiomic features can be categorized into three main groups: (I) geometry, (II) intensity, and (III) texture. Geometric features describe the three-dimensional shape of the tumor, while intensity features reflect the first-order statistical distribution of voxel intensities within the tumor. Texture features, on the other hand, analyze the patterns and the second- and higher-order spatial distributions of these intensities. A total of 1,561 radiomic features were extracted, encompassing first-order features, shape-based features, and a variety of matrix features, including gray level co-occurrence matrix (GLCM) features, gray level dependence matrix (GLDM) features, gray level run length matrix (GLRLM) features, gray level size zone matrix (GLSZM) features, and neighborhood gray-tone difference matrix (NGTDM) features.

### 2.5 Radiomics feature selection

To eliminate differences in index dimensions, Z-score normalization was applied to account for the varying scales of the manually derived radiomic features. Three methods were employed to select the final variables. Initially, the Mann-Whitney U test was performed to filter all radiomic features, retaining only those with a *p*-value of less than 0.05. Subsequently, Pearson’s rank correlation coefficient was computed to evaluate the correlation between features, and those with an intraclass correlation coefficient (ICC) below 0.9 were discarded to guarantee high repeatability. Finally, the least absolute shrinkage and selection operator (LASSO) regression model was employed to identify the final variables for model construction. Ultimately, the best features were incorporated into the prediction models, which were developed using 10-fold cross-validation.

### 2.6 Model development and validation

Three models were developed using the development set of 78 patients: model I (the clinical model), model II (the radiomics model), and model III (the clinical-radiomics model). For radiomics models, we tested 15 machine learning algorithms, with the LightGBM model demonstrating the best performance (**Appendix 1**). However, the clinical-radiomics model was chosen as the nomogram to enhance convenience for clinical application.

The external validation set (34 patients) used to evaluate model performance. The model’s performance was assessed through several metrics, including accuracy, sensitivity, specificity, precision, positive predictive value, negative predictive value, and F1-Score. Additionally, the receiver operating characteristic (ROC) curve was calculated along with the area under the ROC curve (AUC). Calibration was assessed through calibration plots, which depicted the relationship between predicted probabilities and observed proportions. To evaluate the clinical utility and benefits of the predictive model, decision curve analysis (DCA) was conducted.

### 2.7 Statistical analysis

All statistical analyses were performed using Python packages (version 0.13.2). Group differences were evaluated using either Student’s t-test or Mann–Whitney U test for continuous variables, while categorical variables were analyzed using the chi-square test or Fisher’s exact test. Multivariate analysis was conducted to select the final variables. Continuous variables that followed a normal distribution are presented as means ± standard deviations (SDs), whereas non-normally distributed variables are reported as medians ± interquartile ranges (IQRs). And odds ratios (ORs), 95% confidence intervals (CIs), HosmerLemeshow (H-L) test were also calculated. And a *p* value < 0.05 was considered statistically significant.

## 3 Results

### 3.1 Clinical and demographic characteristics

The final cohort comprised 112 patients with advanced epithelial OC. This included the development cohort (n=78), which consisted of 55 patients with R0 resection and 23 patients with non-R0 status, and the external validation cohort (n=34), which included 24 patients with R0 resection and 10 patients with non-R0 status. The comparison between the development and external validation cohorts revealed no significant differences between the two groups, nor within each group (p > 0.05), indicating a reasonable classification. **Table 1** present the baseline characteristics of patients in each cohort. In the multivariate analysis, age (p = 0.031; OR = 1.011, 95% CI: 1.003-1.018), CA125 level (p = 0.002; OR = 1.001, 95% CI: 1.000-1.001), presence of hydrothorax (p = 0.003; OR = 1.174, 95% CI: 1.078-1.279), and maximum tumor diameter (p = 0.031; OR = 1.002, 95% CI: 1.001-1.004) were identified as independent predictors of RT status (**Table 2**).

**Table 1:** Clinical and demographic characteristics of development and validation cohort.

Variables	Development cohort (N=78)			External validation cohort (N=34)		
	R0 (N=55)	Non-R0 (N=23)	*P*	R0 (N=24)	Non-R0 (N=10)	*P*
Age	54.55 ± 9.23	62.13 ± 7.14	<0.01	54.88 ± 9.34	62.10 ± 5.51	0.03
BMI	22.23 ± 3.05	22.64 ± 3.51	0.72	22.08 ± 3.73	24.45 ± 1.89	0.08
NLR	3.07 ± 1.86	3.29 ± 1.82	0.70	3.09 ± 1.88	2.47 ± 1.67	0.46
Perioperative platelet	226.82 ± 82.89	211.04 ± 85.68	0.41	230.00 ± 70.89	175.80 ± 78.42	0.06
Perioperative albumin	45.69 ± 5.48	43.75 ± 4.77	0.09	45.80 ± 5.62	45.37 ± 5.87	0.81
CA125	278.36 ± 163.28	465.04 ± 179.81	<0.01	284.46 ± 136.25	403.10 ± 167.44	0.04
HE-4	285.55 ± 135.99	546.83 ± 183.08	<0.01	318.08 ± 143.04	574.10 ± 184.04	<0.01
MAP score	7.93 ± 2.77	17.83 ± 4.39	<0.01	7.33 ± 2.18	20.80 ± 3.68	<0.01
Maximum tumor diameter	117.25 ± 38.89	141.62 ± 37.31	0.01	119.19 ± 38.81	141.91 ± 23.24	0.10
Arterial pulsatility index	0.31 ± 0.14	0.31 ± 0.12	0.96	0.32 ± 0.14	0.35 ± 0.21	0.63
Resistance index	0.26 ± 0.09	0.28 ± 0.09	0.38	0.27 ± 0.10	0.28 ± 0.13	0.79
End diastolic flow rate	17.09 ± 2.50	16.96 ± 2.09	0.83	16.67 ± 1.88	16.63 ± 2.77	0.97
Peak flow rate	23.07 ± 2.30	23.18 ± 2.02	0.89	22.91 ± 1.97	23.24 ± 2.14	0.67
Average flow rate	19.51 ± 2.22	19.94 ± 1.60	0.40	19.72 ± 1.89	19.48 ± 1.78	0.74
Parity			0.24			0.92
1	4 (7.27)	0		3 (12.50)	1 (10.00)	
2	38 (69.09)	21 (91.30)		17 (70.83)	7 (70.00)	
3	7 (12.73)	2 (8.70)		3 (12.50)	1 (10.00)	
4	5 (9.09)	0		1 (4.17)	1 (10.00)	
5	1 (1.82)	0		0	0	
ASA score			0.14			0.32
1	8 (14.55)	5 (21.74)		8 (33.33)	1 (10.00)	
2	16 (29.09)	1 (4.35)		3 (12.50)	2 (20.00)	
3	10 (18.18)	8 (34.78)		3 (12.50)	2 (20.00)	
4	11 (20.00)	5 (21.74)		8 (33.33)	2 (20.00)	
5	10 (18.18)	4 (17.39)		2 (8.33)	3 (30.00)	
Ascites			0.59			0.13
0	22 (40.00)	3 (13.04)		9 (37.50)	4 (40.00)	
1	19 (34.55)	4 (17.39)		8 (33.33)	3 (30.00)	
2	14 (25.45)	16 (69.57)		7 (29.17)	3 (30.00)	
Hydrothorax			0.01			0.98
0	23 (41.82)	7 (30.43)		4 (16.67)	5 (50.00)	
1	16 (29.09)	9 (39.13)		9 (37.50)	2 (20.00)	
2	16 (29.09)	7 (30.43)		11 (45.83)	3 (30.00)	

A *p* value < 0.05 was considered statistically significant.

ORs, Odds ratios; CIs, Confidence intervals; BMI, Body mass index; NLR, Neutrophil-to-lymphocyte ratio; CA125, Cancer antigen-125; HE-4, Human epididymis protein 4; MAP score, metastases in abdomen and pelvis score; ASA score, American Society of Anesthesiology score.

**Table 2:** the univariate and multivariate logistic regression analysis of development cohort.

Variables	Univariate logistic regression analysis			Multivariate logistic regression analysis		
	OR	OR 95% CI	*P*	OR	OR 95% CI	*P*
Age	1.02	1.01-1.03	0.001	1.01	1.00-1.02	0.030
BMI	1.00	0.98-1.04	0.609			
NLR	1.01	0.97-1.06	0.639			
Perioperative platelet	1.00	1.00-1.00	0.450			
Perioperative albumin	0.99	0.97-1.00	0.145			
CA125	1.00	1.00-1.00	0.000	1.00	1.00-1.00	0.002
HE-4	0.89	0.85-0.93	0.365			
MAP score	0.95	0.94-0.96	0.210			
Maximum tumor diameter	1.00	1.00-1.00	0.013	1.00	1.00-1.00	0.031
Arterial pulsatility index	0.98	0.51-1.90	0.959			
Resistance index	1.65	0.65-4.24	0.376			
End diastolic flow rate	0.96	0.96-1.03	0.830			
Peak flow rate	1.00	0.97-1.05	0.837			
Average flow rate	1.02	0.98-1.07	0.397			
Parity	0.92	0.81-1.04	0.244			
ASA score	1.01	0.95-1.08	0.757			
Ascites	1.24	1.12-1.36	0.000	1.17	1.08-1.28	0.003
Hydrothorax	1.65	0.65-4.24	0.376			

A *p* value < 0.05 was considered statistically significant.

ORs, Odds ratios; CIs, Confidence intervals; BMI, Body mass index; NLR, Neutrophil-to-lymphocyte ratio; CA125, Cancer antigen-125; HE-4, Human epididymis protein 4; MAP score, metastases in abdomen and pelvis score; ASA score, American Society of Anesthesiology score.

### 3.2 Radiomics characteristics

**Figure 1:**
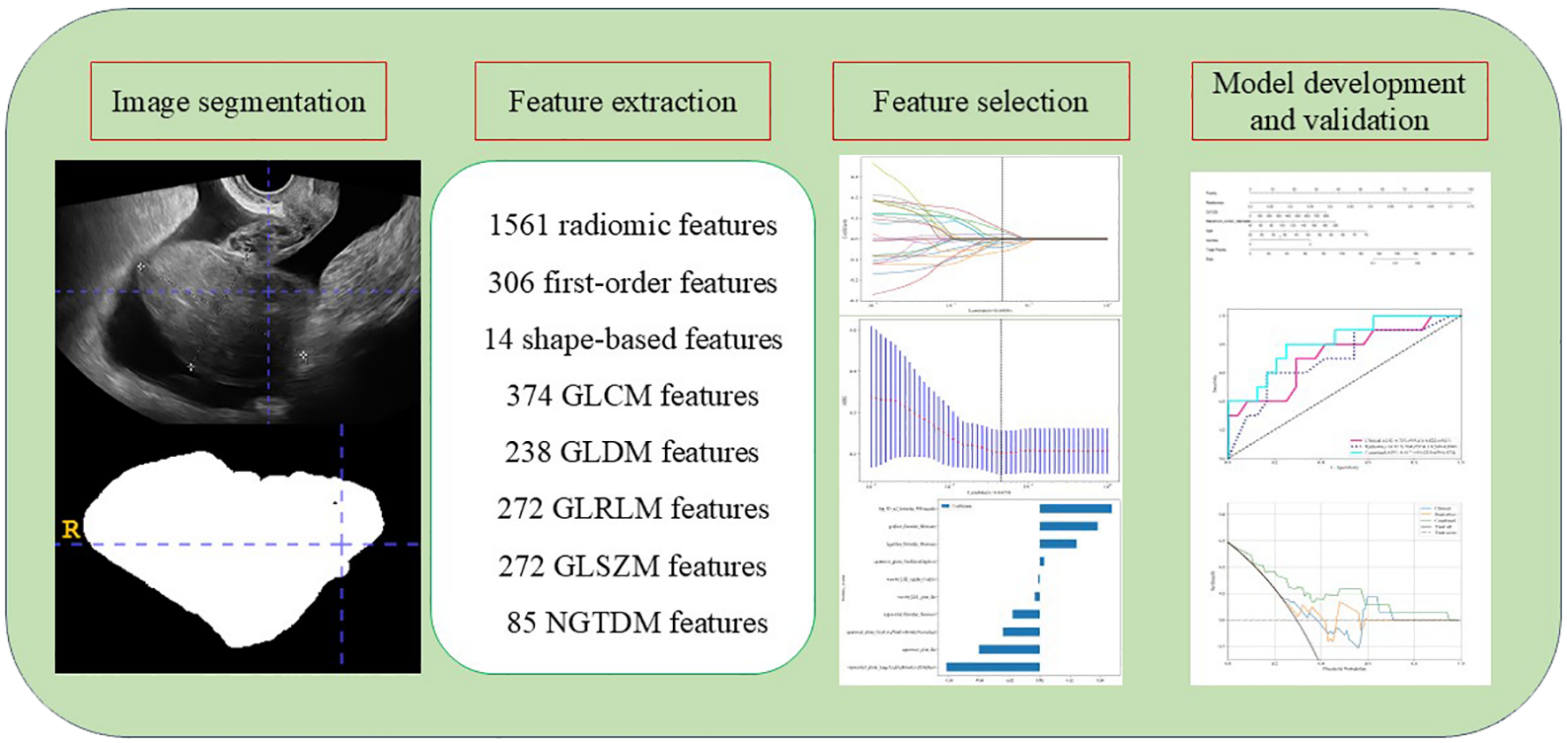
Study flowchart of the radiomics analysis.

**Figure 2:**
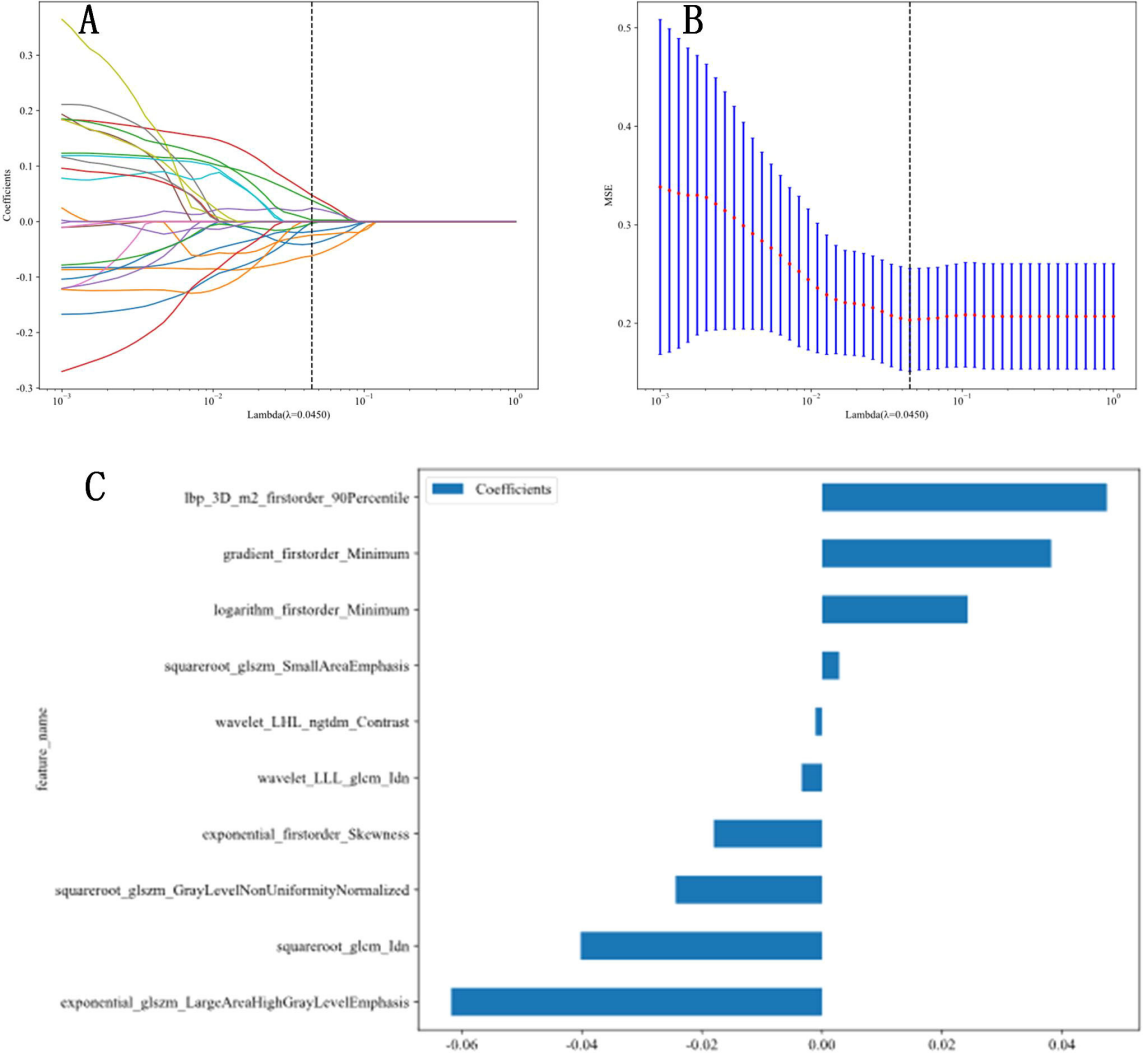
Radiomic feature extraction. **(A, B)** Radiomic features extraction using least absolute shrinkage and selection operator (LASSO) algorithm. **(C)** The final features included in our study.

A total of 1,561 radiomic features were extracted from ultrasound images, which included 306 first-order features, 14 shape-based features, 374 features from the GLCM, 238 features from the GLDM, 272 features from the GLRLM, 272 features from the GLSZM, and 85 features from the NGTDM. The *t*-test or Mann-Whitney U test was utilized for the preliminary screening of all features, resulting in the inclusion of 42 features. Subsequently, Pearson correlation analysis was conducted, revealing 25 features that were significantly different between the two groups. Next, LASSO regression was conducted using 10-fold cross-validation with the minimum criterion to determine the optimal λ values. The λ value that resulted in the lowest cross-validation errors is illustrated in **Figures 1** and **2**. Following this, ten features with nonzero coefficients were used for this task. Finally, ultrasonic radiomic features were established using these 10 features, namely exponential_firstorder_Skewness, exponential_glszm_LargeAreaHighGrayLevelEmphasis, gradient_firstorder_Minimum, lbp_3D_m2_firstorder_90Percentile, logarithm_firstorder_Minimum, squareroot_glcm_Idn, squareroot_glszm_GrayLevelNonUniformityNormalized, squareroot_glszm_SmallAreaEmphasis, wavelet_LHL_ngtdm_Contrast, wavelet_LLL_glcm_Idn (**Figures 1**, **2**).

### 3.3 Model construction and performance assessment

**Figure 3:**
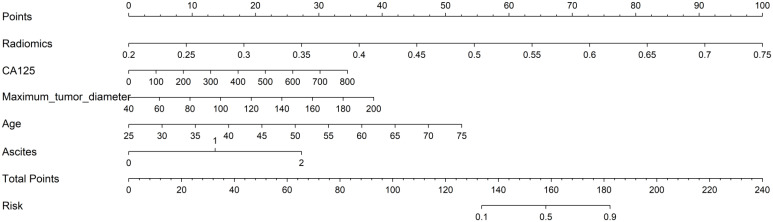
A nomogram integrates clinical parameters and radiomics features.

We developed three models to identify patients suitable for optimal primary debulking surgery. Model 1 (the clinical model) was based solely on clinical characteristics using the LightGBM algorithm. Model 2 (the radiomics model) relied exclusively on ultrasonic radiomics characteristics, also employing the LightGBM algorithm (**Appendix 1**). Model 3 (the clinical-radiomics model) was an integrative nomogram that combined clinical and radiomics features to enhance clinical application convenience (**Figure 3**).

**Figure 4:**
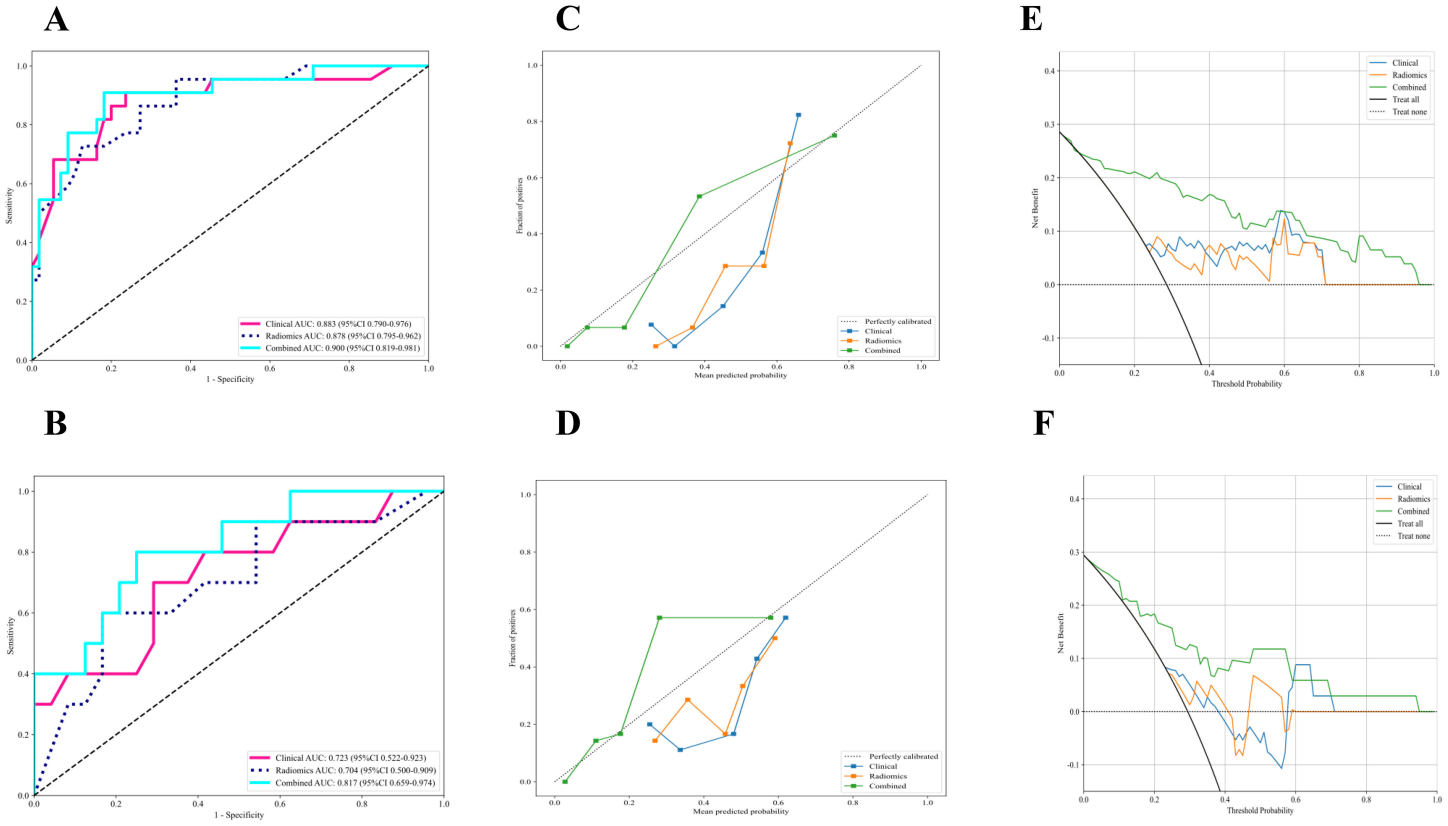
The performance of clinical model, radiomics model and combined nomogram with ROC, calibration curves and decision curve analysis. **(A, B)** ROC curves of each model in the **(A)** development and **(B)** external validation cohort for prediction of RT status. **(C, D)** Calibration curves of each model in the **(C)** development and **(D)** external validation cohort for prediction of RT status, and A 45° diagonal line indicates perfect calibration. **(E, F)** Decision curve analysis of each model in the **(E)** development and **(F)** external validation cohort for prediction of RT status, and the colored lines were expected net benefit of per patient. ROC, Receiver operating characteristics curves; RT, Residual tumor.

The radiomic-clinical nomogram demonstrated superior performance compared to the clinical or radiomics models alone, achieving an accuracy of 0.84, a sensitivity of 0.80, a specificity of 0.75, a precision of 0.88, a positive predictive value of 0.81, a negative predictive value of 0.86, an F1-Score of 0.78, and an AUC of 0.82 in the external validation set (**Table 3**). **Figure 4** illustrates the AUC for both the development and external validation cohorts. The calibration curves for the radiomic-clinical nomogram demonstrated strong agreement between predicted and observed outcomes in both the development and validation cohorts (**Figure 4**). The Hosmer-Lemeshow (HL) test indicated favorable goodness-of-fit for the data (all p > 0.05). Furthermore, the DCA revealed that the nomogram offers greater clinical benefit (**Figure 4**), namely, the DCA for the three models indicates that this new diagnostic approach yields a greater net benefit (where a value greater than 0 indicates patient benefit) in predicting the residual tumor status in patients with advanced OC, with the clinical-radiomics model showing a more significant benefit compared to the clinical model or radiomics model.

**Table 3:** The performance of clinical model, radiomics model and combined nomogram for predicting RT status.

Model	Cohort	AUC	ACC	Sen	Spe	PPV	NPV	Precision	F1
Clinical	Development	0.883	0.883	0.883	0.883	0.883	0.883	0.883	0.883
	Validation	0.723	0.723	0.723	0.723	0.723	0.723	0.723	0.723
Radiomics	Development	0.878	0.878	0.878	0.878	0.878	0.878	0.878	0.878
	Validation	0.704	0.704	0.704	0.704	0.704	0.704	0.704	0.704
Combined	Development	0.900	0.900	0.900	0.900	0.900	0.900	0.900	0.900
	Validation	0.817	0.817	0.817	0.817	0.817	0.817	0.817	0.817

AUC, Area under the curve; ACC, Accuracy; Sen, Sensitivity; Spe, Specificity; PPV, Positive predictive value; NPV, Negative predictive value; F1, F1-Score.

## 4 Discussion

In our study, we integrated primary radiomic features, laboratory findings, and clinical factors from patients with advanced epithelial OC to create and validate a radiomics-clinical nomogram. This nomogram is designed for individualized preoperative prediction of treatment response (RT) status. The results demonstrated that the integrated radiomic-clinical nomogram showed enhanced predictive performance compared to using radiomic or clinical signatures individually after external validation. The final model is capable of identification of the RT status prior to surgery. This advancement enhances clinical decision-making, patient communication, and prognosis assessment. For those with a low probability of attaining R0 resection, the surgical intervention should be avoided if incomplete resection. The presence or absence of response to treatment (RT) following PDS or IDS is the most significant factor influencing the prognosis of patients with advanced OC. Notably, a 10% increase in the rate of complete tumor resection can lead to a 5% improvement in overall survival for these patients (15). Research has shown that RT status is an independent and significant prognostic factor for patients with advanced OC. The extent of RT is inversely correlated with patient survival, disease-free survival (DFS), and overall survival (OS) (5, 16). According to Kehoe et al., patients with OC who underwent PDS followed by RT excision experienced the most favorable prognosis (17). High-grade serous ovarian cancer (HGSOC) is the most common and aggressive histological subtype of OC, and complete resection of all visible lesions (RT-resection) in advanced HGSOC patients after PDS is linked to the best outcomes (5, 18). Therefore, it is essential to assess all epithelial OC patients suspected of being at stage IIIC or IV to determine their eligibility for PDS prior to initiating therapy, in line with the clinical practice guidelines set forth by the Society of Gynecologic Oncology and the American Society of Clinical Oncology (19).

For patients in whom achieving satisfactory tumor reduction is challenging, neoadjuvant chemotherapy should be considered prior to PDS. Kevin et al. (21) demonstrated that the mean tumor nuclear area and the major axis length of the stroma are significant factors that can improve risk stratification in patients with HGSOC. For the ultrasonic radiomic characteristics, three methods were employed to select the final variables, resulting in the inclusion of 10 features from a total of 1,561 radiomic features in our model, effectively eliminating invalid variables. Previous studies have demonstrated that all ultrasonic radiomics and clinical features included in our study are relevant to the diagnosis, treatment, and prognosis of ovarian cancer (15, 18, 21, 24).

CA-125 is one of the most commonly used serum biomarkers for OC. Some studies (13, 20) have found that preoperative CA-125 levels can predict gross residual disease at PDS for advanced epithelial OC. Additionally, moderate to severe ascites has been associated with residual disease (13) and may serve as a surrogate indicator of advanced disease across multiple anatomic locations. The maximum tumor diameter is a critical predictor for individualized preoperative assessment of RT status in patients with advanced OC, as reflected in radiomic shape-based features. For patients who are unlikely to achieve satisfactory tumor reduction, neoadjuvant chemotherapy should be considered prior to PDS. Kevin et al. (21) demonstrated that the mean tumor nuclear area and the major axis length of the stroma are important factors for improving risk stratification in patients with HGSOC. In analyzing ultrasonic radiomic characteristics, three methods were utilized to select the final variables, resulting in the inclusion of 10 features from a total of 1,561 radiomic features in our model, effectively eliminating invalid variables.

Ultrasound offers several advantages, including real-time display, convenience, and affordability, making it widely used for screening and preoperative evaluation of OC. Recently, applications of ultrasound-based radiomics have been reported in tumor diagnosis (12), pathology grading (22), vascular invasion assessment, therapeutic evaluation (23), and prognostic prediction (24). However, there are few reports on RT status based on ultrasonics. Meanwhile, several radiomic models for predicting RT status based on computed tomography (CT) and magnetic resonance imaging (MRI) have been developed and validated (25, 26). Lu et al. (26) developed an MRI-based radiomic-clinical nomogram that successfully predicted RT status preoperatively in patients with HGSOC. A multicenter assessment was conducted to evaluate the efficacy of preoperative CT scans and CA-125 levels in predicting gross residual disease following PDS for advanced epithelial OC (25). However, the pelvic CT-based model was primarily developed with a focus on abdominal metastases. These findings support the hypothesis that radiomic features can effectively predict treatment response (RT) status by capturing variations in tumor heterogeneity.

There are several limitations to our study. Firstly, it relies on a small sample size, necessitating larger databases and multicenter studies to confirm the generalizability of this model. Second, future studies should integrate CT or contrast-enhanced CT and MRI or contrast-enhanced MRI into the predictive model to enhance the prediction of RT status in OC. Finally, our study focused exclusively on advanced epithelial OC subtypes, excluding rare variants. Future research should include data from additional OC subtypes to improve the models’ universality and clinical applicability.

## 5 Conclusion

In our study, we confirmed the clinical value of ultrasound-based radiomics for the preoperative prediction of treatment response (RT) status in patients with advanced epithelial OC, and radiomic feature extraction and selection may provide a deeper understanding of ultrasound imaging mechanism. The comprehensive model combined clinical and ultrasonic radiomics features not only had a better performance in preoperative identification of complete resection of all visible diseases but also had a higher generalization ability.

## Data Availability

The original contributions presented in the study are included in the article/**Supplementary Material**. Further inquiries can be directed to the corresponding authors.
