# Supplementary material for: Ultrasonic radiomics-based nomogram for preoperative prediction of residual tumor in advanced epithelial ovarian cancer: a multicenter retrospective study
Source: Front Oncol. 2025 Feb 4;15:1540734. doi: 10.3389/fonc.2025.1540734 (PMC11832395; doi:10.3389/fonc.2025.1540734)
Supplement: Supplementary file 1 [file DataSheet1.docx]

# This is an example of settings that can be used as a starting point for analyzing US data. This is only intended as a

# starting point and is not likely to be the optimal settings for your dataset. Some points in determining better values

# are added as comments where appropriate

# When adapting and using these settings for an analysis, be sure to add the PyRadiomics version used to allow you to

# easily recreate your extraction at a later timepoint:

# ############################# Extracted using PyRadiomics version: <version> ######################################

imageType:

Original: {}

LoG:

sigma: [1.0, 2.0, 3.0] # If you include sigma values >5, remember to also increase the padDistance.

Wavelet: {}

LBP3D: {}

Exponential: {}

Square: {}

SquareRoot: {}

Logarithm: {}

Gradient: {}

featureClass:

# redundant Compactness 1, Compactness 2 an Spherical Disproportion features are disabled by default, they can be

# enabled by specifying individual feature names (as is done for glcm) and including them in the list.

shape:

firstorder:

glcm: # Disable SumAverage by specifying all other GLCM features available

- 'Autocorrelation'

- 'JointAverage'

- 'ClusterProminence'

- 'ClusterShade'

- 'ClusterTendency'

- 'Contrast'

- 'Correlation'

- 'DifferenceAverage'

- 'DifferenceEntropy'

- 'DifferenceVariance'

- 'JointEnergy'

- 'JointEntropy'

- 'Imc1'

- 'Imc2'

- 'Idm'

- 'Idmn'

- 'Id'

- 'Idn'

- 'InverseVariance'

- 'MaximumProbability'

- 'SumEntropy'

- 'SumSquares'

glrlm:

glszm:

gldm:

ngtdm:

setting:

# Normalization:

# most likely not needed, US gray values reflect absolute world values (HU) and should be comparable between scanners.

# If analyzing using different scanners / vendors, check if the extracted features are correlated to the scanner used.

# If so, consider enabling normalization by uncommenting settings below:

normalize: true

normalizeScale: 1000 # This allows you to use more or less the same bin width.

# Resampling:

# Usual spacing for US is often close to 1 or 2 mm, if very large slice thickness is used,

# increase the resampled spacing.

# On a side note: increasing the resampled spacing forces PyRadiomics to look at more coarse textures, which may or

# may not increase accuracy and stability of your extracted features.

interpolator: 'sitkNearestNeighbor'

resampledPixelSpacing: [3, 3, 3]

padDistance: 10 # Extra padding for large sigma valued LoG filtered images

# Mask validation:

# correctMask and geometryTolerance are not needed, as both image and mask are resampled, if you expect very small

# masks, consider to enable a size constraint by uncommenting settings below:

#minimumROIDimensions: 2

#minimumROISize: 50

correctMask: true

# Image discretization:

# The ideal number of bins is somewhere in the order of 16-128 bins. A possible way to define a good binwidt is to

# extract firstorder:Range from the dataset to analyze, and choose a binwidth so, that range/binwidth remains approximately

# in this range of bins.

binWidth: 5

# first order specific settings:

voxelArrayShift: 1000 # Minimum value in HU is -1000, shift +1000 to prevent negative values from being squared.

# Misc:

# default label value. Labels can also be defined in the call to featureextractor.execute, as a commandline argument,

# or in a column "Label" in the input csv (batchprocessing)

# label: 1
